# MicroRNA-1269a Promotes Proliferation and Arrest of Apoptosis of Glioma Cells by Directly Targeting ATRX

**DOI:** 10.3389/fonc.2020.563901

**Published:** 2020-10-29

**Authors:** Yulian Zhang, Qi Wang, Na Luo, Jiang Liu, Hongxiang Ren, Xu Shao, Li Zhang, Yanbing Yu

**Affiliations:** ^1^Department of Neurosurgery, Peking University China-Japan Friendship School of Clinical Medicine, Beijing, China; ^2^Department of Neurosurgery, China-Japan Friendship Hospital, Beijing, China; ^3^Department of Neurosurgery, Graduate School of Peking Union Medical College, Beijing, China

**Keywords:** miR-1269a, ATRX, glioma, target, prognosis, proliferation, apoptosis

## Abstract

Glioma is one of the deadliest malignant brain tumors in adults worldwide. MicroRNA (miR) has been reported to be a pivotal regulator in human tumors. The aim of this study was to determine the expression, function, and mechanism of action of miR-1269a in glioma progression. The expression of miR-1269a was higher in both glioma cases reported in databases and glioma cell lines, and it was highly associated with poorer prognosis. Next, it was shown *in vitro* that mimic of miR-1269a could promote glioma progression and arrest apoptosis, whereas the inhibition of miR-1269a exhibited the opposite effects. In addition, miR-1269a was found to directly target ATRX chromatin remodeler by a dual-luciferase reporter assay. Moreover, ATRX overexpression could reverse the suppressive effects of miR-1269a on proliferation and apoptosis *in vitro*. *In vivo* subcutaneous xenograft tumor assay was also performed to confirm the phenotypes and molecular mechanism involved. Taking the findings together, our study implies that the miR-1269a/ATRX axis is a novel therapeutic target of glioma.

## Introduction

Glioma is one of the deadliest malignant brain tumors in adults worldwide. Owing to its high invasiveness and the unclear boundary with normal brain tissue, it is difficult to remove completely, and recurrence readily occurs (1). Glioblastoma (GBM) is the type of glioma most resistant to conventional radiotherapy, chemotherapy, and other comprehensive treatment methods (2). Therefore, in-depth study of the molecular mechanisms behind the pathogenesis of glioma and the search for key molecular biological targets in its occurrence and development may provide important concepts and evidence for new treatment methods (3).

MicroRNAs (miRNAs) are small, non-coding, highly conserved endogenous RNA molecules that are involved in regulating the expression of target mRNAs. Previous studies reported that miR-1269a is expressed at a high level in colorectal cancer (4), hepatocellular carcinoma (5), lung cancer (6), and gastric cancer (7) and functions as a tumor promoter. However, the expression and biological roles of miR-1269a in glioma remain unknown.

To date, our results have shown that miR-1269a is expressed at a higher level in GBM based on results reported in databases and glioma cell lines. Upregulated miR-1269a was also shown to be significantly associated with proliferation and apoptosis in glioma cell lines. Next, *in vivo* results showed that increased miR-1269a expression significantly promoted tumor growth. Furthermore, we found that miR-1269a directly targets ATRX chromatin remodeler *in vitro* and *in vivo*. Our study identified that miR-1269a/ATRX is a potential therapeutic target for the treatment of glioma.

## Methods

### Database Analysis

Bioinformatics analysis was performed using The Cancer Genome Atlas (TCGA, https://cancergenome.nih.gov/) database, Chinese Glioma Genome Atlas (8, 9) (CGGA, http://www.cgga.org.cn/), and Gene Expression Omnibus database (GEO, https://www.ncbi.nlm.nih.gov/geo/). To compare the difference of miRNA expression between normal and GBM samples in databases, we searched the NCBI's GEO database for miRNA GBM chips containing normal brain tissue samples (keywords: glioblastoma, normal brain, miRNA profiling); we got GSE25632 (containing 82 primary GBM tissues and 5 normal brain tissues) and GSE103229 (containing 5 GBM tissues and 5 normal brain tissues). To compare the difference of miRNA expression between primary and recurrent GBM tissues, we searched the GEO database and got GSE32466 (containing 12 paired primary and recurrent GBM tissues). The datasets were all downloaded from the GEO database. The thresholds of differential expressed miRNAs were set as logFC >1.5, or < -1.5, and *p* < 0.05. ATRX protein expression that differed between glioma and normal brain tissues as revealed by immunohistochemistry (IHC) was obtained from the Human Protein Atlas (HPA) (10). The staining intensity evaluation method quoted from HPA: Protein expression score based on IHC is manually scored with regard to staining intensity (negative, weak, moderate, or strong) and fraction of stained cells (<25%, 25–75%, or >75%). Each combination of intensity and fractions is automatically converted into an protein expression level score as follows: negative—not detected; weak <25%—not detected; weak combined with either 25–75% or 75%—low; moderate <25%—low; moderate combined with either 25–75% or 75%—medium; strong <25%—medium, strong combined with either 25–75% or 75%—high (https://www.proteinatlas.org).

### Cell Lines and Culture

U251, SNB19, SHG44, A172, HEB (human embryonic brain cells), and HEK293 were purchased from the Cell Bank of the Chinese Academy of Sciences. HEB cells were isolated from embryonic brain cells and maintain neuronal and glial phenotypes (11). The U251, SNB19, SHG44, A172, HEB, and HEK293 cells were cultured in DMEM (Invitrogen, Thermo Fisher Scientific, USA). All culture media contained 10% fetal bovine serum (Gibco, NY, USA) supplemented with penicillin streptomycin and non-essential amino acids (Gibco, NY, USA). The above cells were cultured in a humidified incubator at 37°C with an atmosphere of 5% CO_2_ and 95% air.

### Cell Transfection

To overexpress or inhibit the expression of miR-1269a in cells, we used a mimic and inhibitor of miR-1269a obtained from GenePharma Co. Ltd. (Shanghai, China). The sequences were as follows: miR-1269a mimic, 5′-CUGGACUGAGCCGUGCUACUGG-3′; mimic negative control (NC), 5′-UCCCAUCGGACUAAAUCUGCGAA-3′; miR-1269a inhibitor, 5′-CCAGUAGCACGGCUCAGUCCAG-3′; and inhibitor NC, 5′-UUCGCAGAUUUAGUCCGAUGGGA-3′. Besides, the pcDNA3.1 plasmid was used for target gene overexpression. The ATRX overexpression plasmid pcDNA3.1-ATRX OE was constructed and obtained from the (Clontech, CA, USA). The cells were seeded into six-well plates 24 h prior to transfection. The cells were transfected with Lipofectamine 3000 (Invitrogen, Carlsbad, CA, USA) when the cell density reached approximately 50%−70%.

### RNA Extraction and Quantitative Polymerase Chain Reaction

Total RNA was extracted from cell samples using TaKaRa MiniBEST Universal RNA Extraction Kit (TaKaRa, Tokyo, Japan) and was converted into complementary DNA (cDNA) using TaKaRa PrimeScript II 1^st^ Strand cDNA Synthesis Kit (TaKaRa, Tokyo, Japan). Small RNAs were isolated using miRcute miRNA isolation kit (Tiangen Biotech, Beijing, China) and converted into cDNA using Mir-X^TM^ miRNA First-Strand Synthesis Kit (TaKaRa, Tokyo, Japan) or with specific primers. Quantitative polymerase chain reaction (qPCR) was performed using QuantiNova SYBR Green PCR Kit (QIAGEN, Hilden, Germany). The protocol of real-time qPCR was as follows: initially 95°C for 2 min, followed by 40 cycles of 95°C for 5 s and 60°C for 15 s. The sequences of the primers used are displayed in Table 1. Quantifications were normalized by taking GAPDH or U6 as an endogenous control and were calculated by the 2^−ΔΔCt^ method and purchased from TsingKe Biological Technology (Beijing, China). All experiments were repeated in triplicate.

**Table 1:** The sequences of the primers used in this study.

**Primer**	**5^**′**^-3^**′**^**
GAPDH forward primer	ACAGCCTCAAGATCATCAGC
GAPDH reverse primer	GGTCATGAGTCCTTCCACGAT
ATRX forward primer	GCAACCTTGGTCGAAAGGAGT
ATRX reverse primer	GGCTCTGGGTGACAAATGTAG
miR-1269a -RT primer	GTCGTATCCAGTGCGTGTCGT GGAGTCGGCAATTGCAC TGGATACGACCCAGTA
miR-1269a forward primer	CTGGACTGAGCCGTGC
miR-1269a reverse primer	CAGTGCGTGTCGTGGA

*GAPDH and U6 served as endogenous controls for mRNA or miRNA, respectively*.

### Western Blot

Western blot was performed in cultured cells as indicated. The cells were prepared from cell lines with RIPA lysis buffer kit (Boster Biological Tech, Wuhan, China), and the total protein was quantified using the bicinchoninic acid method (Thermo Fisher Scientific, Waltham, MA, USA). Whole-cell proteins (25 μg) were separated using 10% sodium dodecyl sulfate–polyacrylamide gel electrophoresis. Subsequently, the gels were transferred to polyvinylidene fluoride membranes (Beyotime, Shanghai, China). Then, the membranes were blocked with 5% skim milk in TBST for 1 h at room temperature. After that, the membranes were incubated with the primary antibodies: GAPDH (1:6,000; Proteintech, USA), Ki67 (1:1,000; Abcam, MA, USA), Bax (1:1,000; Cell Signaling, Germany), Bcl2 (1:3,000; Proteintech, USA), cleaved caspase3 (1:1,000; Cell Signaling, Germany), caspase3 (1:1,000; Cell Signaling, Germany), and ATRX (1:2000; Proteintech, USA) overnight at 4°C. After washing with TBST buffer three times, membranes were reacted with the secondary goat anti–rabbit/mouse antibody conjugated with horseradish peroxidase for 1 h at room temperature. Signals were measured using enhanced chemiluminescence) kit (MD Millipore, Germany) in the Bio-Rad ChemiDocXRS Imaging system (Bio-Rad, Hercules, CA, USA).

### Cell Proliferation Assay

The proliferation capacities of SHG44 and U251 cells after transfection were measured using the Cell Counting Kit-8 (CCK8) assay (Dojindo, Japan). Cell suspensions at a density of 3,000 cells were plated in a 96-well plate. At 0, 24, 48, and 72 h after transfection, CCK-8 (10 μL) reagent was added into each well. Then, the plate was put back into an incubator for 1 h; finally, the optical density of each well was measured at 450 nm. All experiments were performed three times.

### Cell Apoptosis Assay

Cell apoptosis assay was performed with annexin V–fluorescein isothiocyanate (FITC)/propidium iodide (PI) detection kit (KeyGEN BioTECH, JiangSu, China) following the manufacturer's instructions. The cells were trypsinized and washed with phosphate-buffered saline. Next, the cells were resuspended with binding buffer and incubated with annexin V–FITC and PI, and then maintained in the dark for 10 min. After 10 min of incubation at room temperature, the cells were immediately analyzed by FACSCanto (BD Bioscience). All experiments were performed three times.

### Luciferase Assay

Direct target genes of miR-1269a were predicted based on TargetScan (http://www.targetscan.org/vert_72/), miRDIP (12), and miRDB (13). The luciferase plasmid was constructed, and the 3′ UTR sequence of ATRX (ATRX-wt) or a mutant sequence (ATRX-mut) was cloned into psiCHECK-2 vector (Promega). 293t cells were seeded into 6-well plates at a density of 2.5 × 10^5^ per well. Cells were transfected with or without miR-1269a mimics/inhibitor or miR-NC and luciferase reporter plasmid using Lipofectamine 3000 (Invitrogen). At 48 h after transfection, the firefly luciferase and Renilla signals were measured using the Dual-Luciferase Reporter Assay Kit (Beyotime, Shanghai, China). For data analysis, the firefly luciferase activity was normalized to Renilla luciferase activity. All experiments were performed three times.

### Xenograft Assay

Six-week-old nude mice were purchased from Shanghai Experimental Animal Center (Shanghai, China) and kept in an SPF animal facility. All animal experiments were conducted in accordance with the institutional ethical and safety guidelines (Institutional Animal Welfare and Ethics Committee, China-Japan Friendship Hospital, Beijing, China). Twelve nude mice were randomly divided into a miR-1269a expression group and an NC group (*n* = 6 per group). U251 cells stably transfected with miR-1269a expression lentivirus (pLVX-miR-1269a) or empty control lentivirus (pLVX vector) were injected into the subaxillary subcutaneous of the nude mice (5 × 10^6^ cells), respectively. Subcutaneous tumor length and width were monitored 15 days after injection, which was repeated every 5 days. Tumor volume was measured as length × width^2^/2. Five weeks after injection, the mice were sacrificed, and the tumors were dissected and weighed.

### Statistical Analysis

All the experimental data were analyzed using GraphPad Prism 7.0 (GraphPad Inc., CA, USA), and data are presented as mean ± standard deviation. Comparisons between two groups (paired) were performed by Student *t* test (paired *t* test). Survival analysis based on TCGA and CGGA was performed using the Kaplan–Meier method. Correlation between miR-1269a and ATRX expression was performed by Spearman correlation analysis. *P* < 0.05 was considered statistically significant.

## Results

### High Expression of miR-1269a Is Associated With Poor Prognosis of Glioma Patients in Datasets

**Figure 1:**
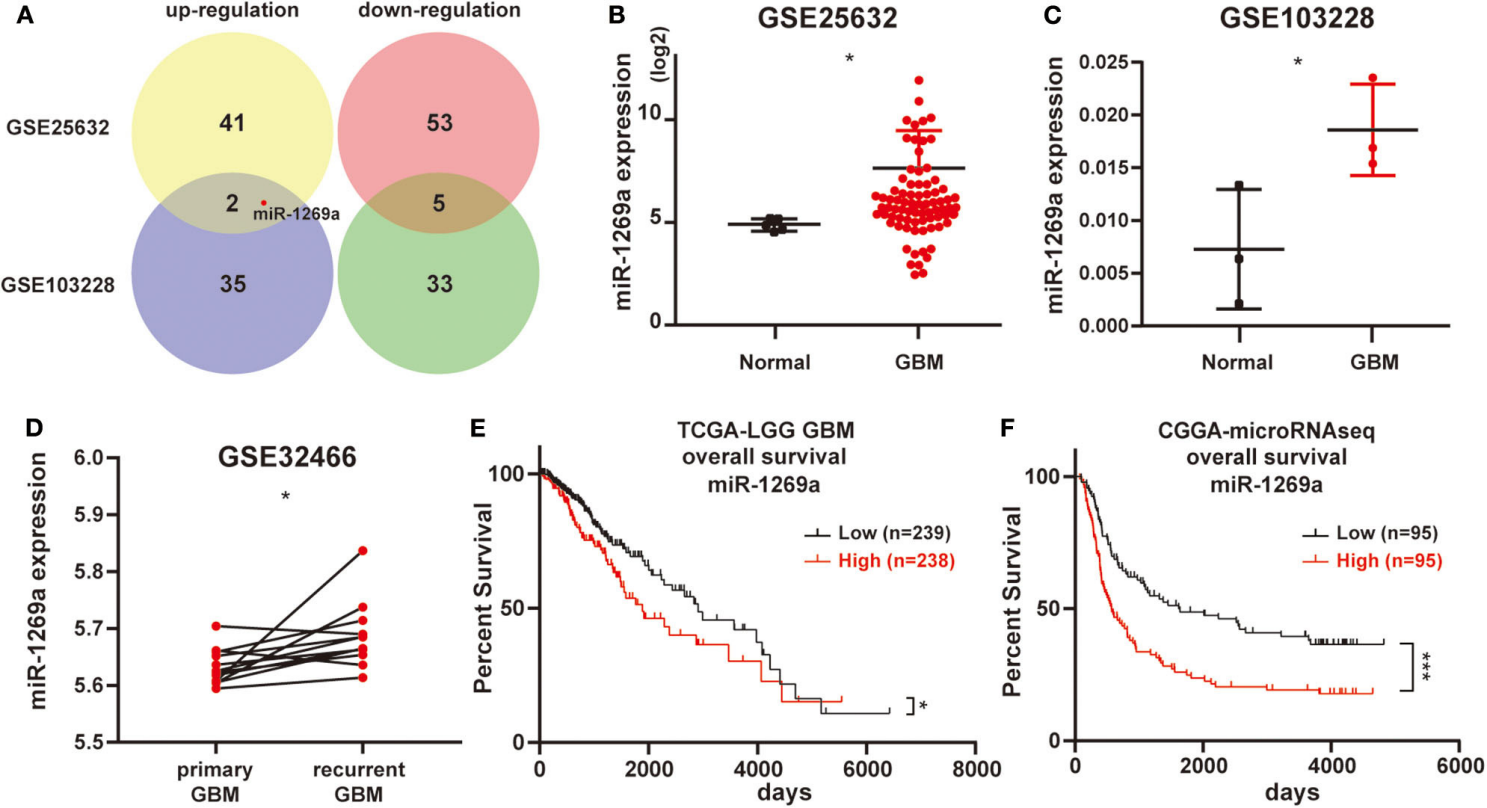
High levels of miR-1269a expression are associated with poor prognosis of glioma patients in datasets. **(A)** The miRNA microarray of glioblastoma samples was obtained from the Gene Expression Omnibus database (GSE25632 and GSE103228). **(B–D)** The expression levels of miR-1269a in glioma based on the GSE databases. **(E,F)** Overall survival according to high or low miR-1269a expression levels in the TCGA and CGGA databases. The median value was used as the boundary of the high and low expression levels of miR-1269a. All data are shown as mean ± SD. *P < 0.05, and ***P < 0.001.

First, we analyzed the miRNA microarray which contains GBM and normal samples in GEO database. Then we got GSE25632 (containing 82 primary GBM tissues and 5 normal brain tissues) and GSE103229 (containing 5 GBM tissues and 5 normal brain tissues). We obtained 101 (GSE25632) and 75 (GSE103229) miRNAs with significantly different expressions, respectively. Further bioinformatics analysis suggested that the expression level of miR-1269a was significantly increased in GBM (all *P* < 0.05; Figures 1A–C). Next, the GSE32466 database, which contained paired GBM samples, was used to confirm these results; the findings showed that the level of miR-1269a was significantly higher in recurrent tissues (*P* = 0.018) (Figure 1D). Furthermore, the survival data in databases indicated that glioma patients with a high miR-1269a level had a significantly poorer overall survival than those with low miR-1269a expression in TCGA low-grade glioma (LGG) database (*P* = 0.024) and CGGA database (*P* = 0.0003) (Figures 1E,F). Therefore, miR-1269a overexpression was a potential indicator for poor prognosis of glioma patients.

### miR-1269a Overexpression Promotes Proliferation and Inhibits Apoptosis in Glioma Cells

**Figure 2:**
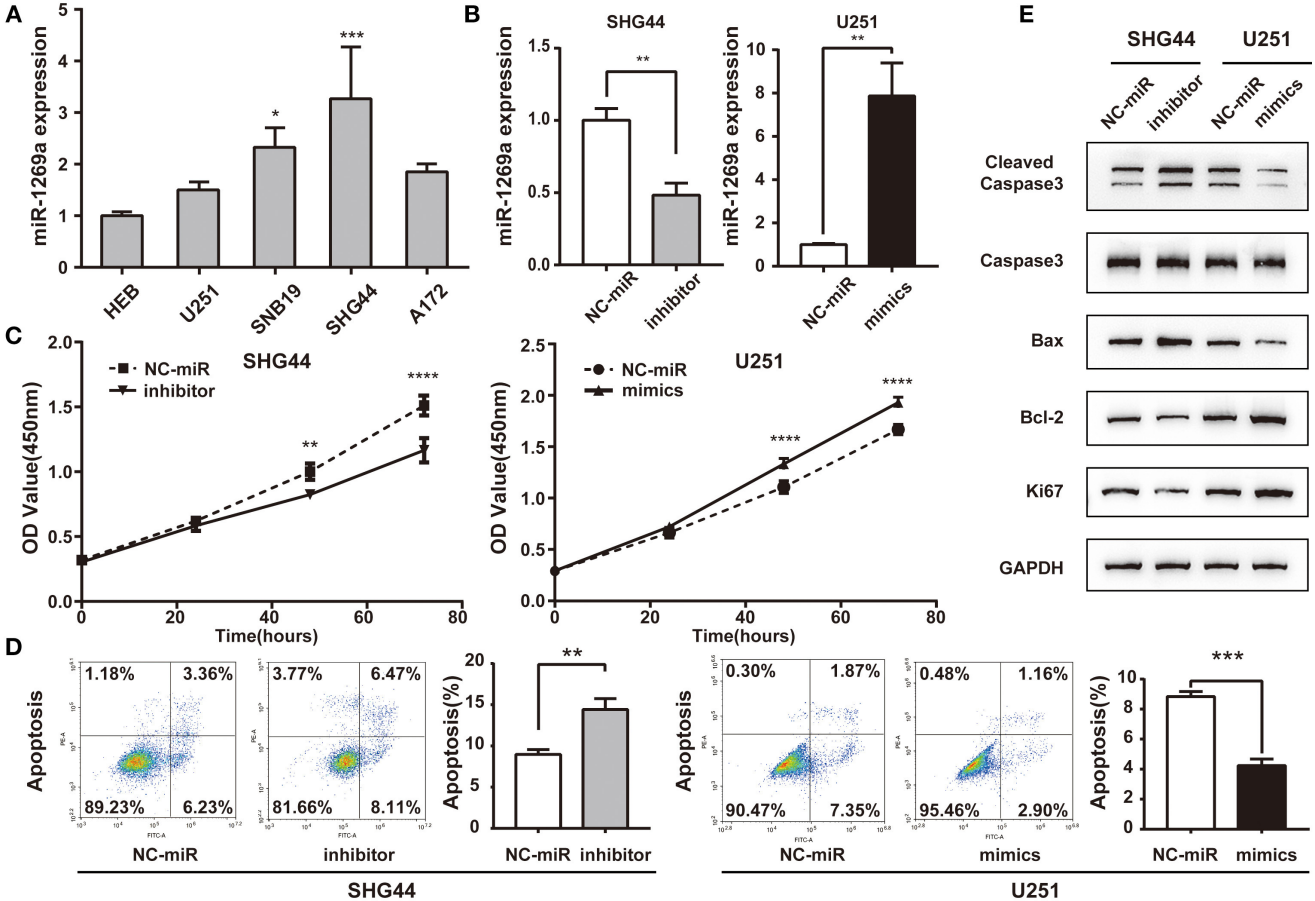
miR-1269a is overexpressed and promotes proliferation and inhibits apoptosis in glioma cells. **(A)** The relative expression levels of miR-1269a in five human glioma cell lines (U251, SNB19, SHG44, and A172) and HEB cell line. **(B)** Transfection efficiency of the miR-1269a inhibitor in SHG44 and mimics in U251 was determined via qPCR. **(C)** Cell proliferation was determined via the CCK8 assay. **(D)** Cell apoptosis was determined via the flow cytometry assay. **(E)** The protein expression levels of proliferation (Ki67) and apoptosis (cleaved caspase 3, caspase 3, Bax, and Bcl-2) were detected using Western blot. *P < 0.05, **P < 0.01, ***P < 0.001, and ****P < 0.0001.

To explore miR-1269a expression in glioma, we first assessed its expression levels in four human glioma cell lines (U251, A172, SNB19, and SHG44) and HEB. As shown in Figure 2A, the expression of miR-1269a was significantly increased in SNB19 and SHG44 cell lines (*P* < 0.05) and slightly increased in U251 and A172 compared with that in the HEB cell line. To further investigate the potential function of miR-1269a in glioma, we transfected SHG44 cells with miR-1269a inhibitor to decrease miR-1269a expression; we also transfected U251 cells with miR-1269a mimic to increase miR-1269a expression. Reverse transcriptase (RT)–qPCR was used to evaluate the expression efficiency. As displayed in Figure 2B, the expression of miR-1269a was markedly decreased by the inhibitor in SNB19 cells and greatly increased by the mimic in U251 cells (all *P* < 0.05).

Then, the CCK-8 assay was used to investigate the role of miR-1269a in glioma cell growth and proliferation. The results showed that miR-1269a inhibitor significantly inhibited the proliferation of SHG44 cells (*P* < 0.0001), whereas miR-1269a mimic dramatically promoted the growth of U251 cells (*P* = 0.001; Figure 2C). Flow cytometry assays were used to measure the apoptosis in U251 and SHG44 cells. The results showed that apoptosis in the miR-1269a inhibitor group was significantly upregulated compared with that in the NC-miR group (*P* = 0.0027), whereas apoptosis in the miR-1269a mimic group was significantly suppressed compared with that in the NC-miR group (*P* = 0.0001; Figure 2D). Western blot was also used to detect the biomarkers of the proliferation-related gene Ki67 and apoptosis-related genes, including cleaved caspase3, caspase3, Bax, and Bcl-2. The results demonstrated that, compared with the findings in the NC-miR group, miR-1269a mimic could promote proliferation and inhibit apoptosis, whereas miR-1269a inhibitor could arrest proliferation and upregulate apoptosis (Figure 2E). Thus, miR-1269a promotes proliferation and inhibits apoptosis in glioma cells.

### ATRX Is a Direct Target of miR-1269a and Is Correlated With Poor Characteristics and Prognosis of Glioma Patients in Datasets

**Figure 3:**
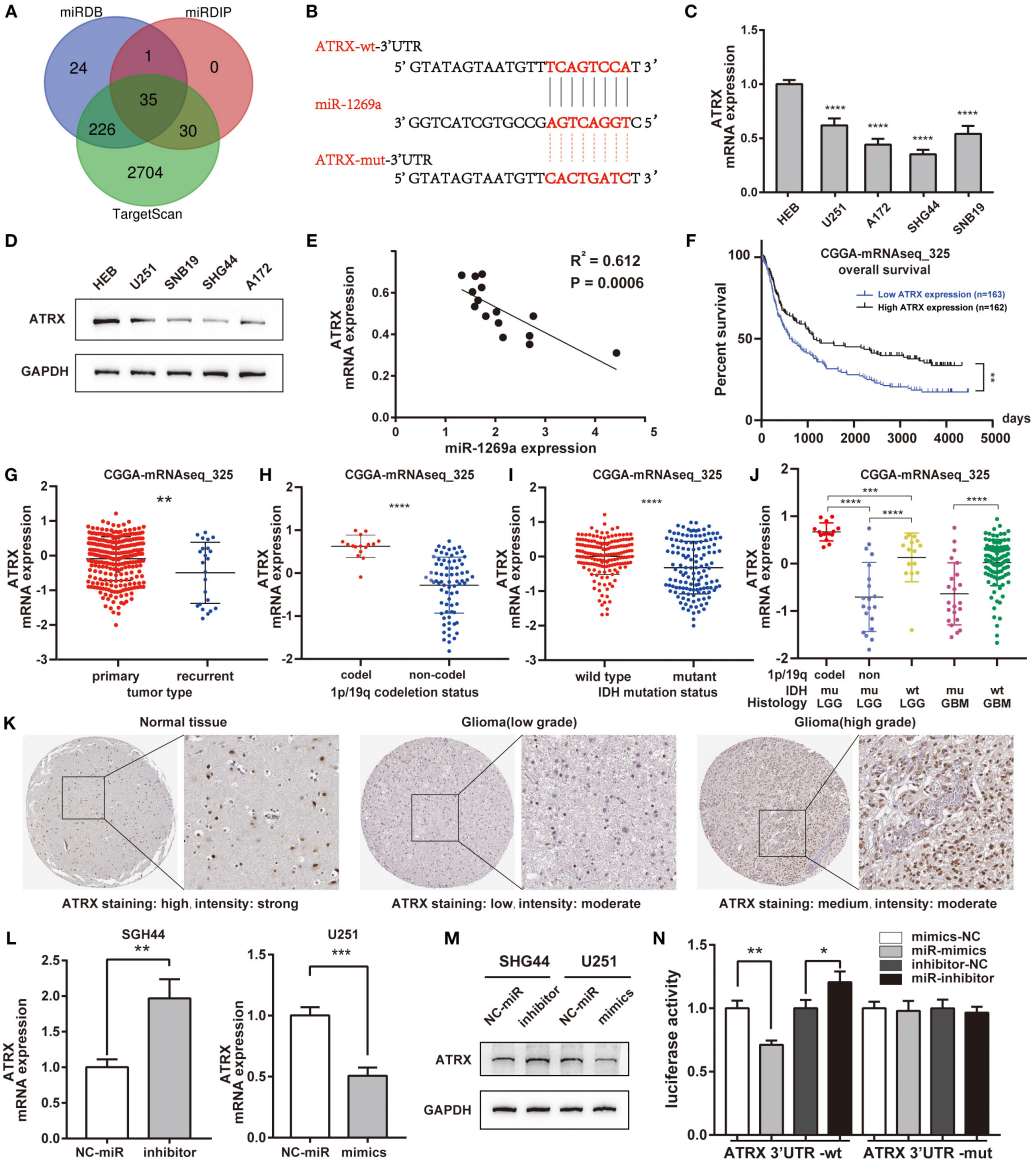
ATRX is a direct target of miR-1269a and is correlated with poor characteristics and prognosis of glioma patients in datasets. **(A)** The direct target genes predicted by TargetScan, miRDIP, and miRDB are shown with a Venn diagram. **(B)** The binding site of miR-1269a and the 3′ UTR of ATRX as predicted by TargetScan. **(C,D)** The mRNA and protein expression levels of ATRX in U251, A172, SHG44, SNB19, and HEB were detected by qPCR and Western blot. **(E)** The expression levels of miR-1269a negatively correlated with those of ATRX mRNA. **(F)** The survival data in the CGGA database indicated that glioma patients with a low level of ATRX expression had a significantly worse overall survival compared with those with high level of ATRX expression. **(G–J)** Low ATRX expression positively correlated with tumor type (recurrent), IDH status (mutant), and 1p/19q non-codeletion. **(K)** Immunohistochemistry results obtained from the HPA database showed that ATRX was at low staining in glioma tissues but was heavily stained in normal brain tissues and was mainly localized in the nucleus of the cells. **(L,M)** The mRNA and protein expression levels of ATRX in a U251 cell with mimics and in an SHG44 cell with miR-1269a inhibitor were detected via qPCR and Western blot. **(N)** The targeting relationship was observed between miR-1269a and ATRX by the dual-luciferase reporter assay. *P < 0.05, **P < 0.01, ***P < 0.001, and ****P < 0.0001.

To further understand the underlying molecular mechanisms of the effect of miR-1269a in glioma, we detected the potential direct target gene of miR-1269a using TargetScan, miRDIP, and miRDB (Figure 3A). The results showed that ATRX might be a target gene of miR-1269a; the binding site of miR-1269a and the 3′ UTR of ATRX is shown in Figure 3B. The mRNA and protein expression levels in glioma cell lines were determined by qPCR and Western blot (Figures 3C,D). The results showed that ATRX was significantly decreased in U251, SNB19, A172, and SHG44 (all *P* < 0.0001) and was negatively correlated with the expression levels of miR-1269a in cell lines (*R*^2^ = 0.612, *P* = 0.0006, Figure 3E). Next, we investigated the ATRX expression in the CGGA database (mRNAseq_325). The survival data indicated that glioma patients with a low ATRX level had significantly poorer overall survival than patients with a high level of ATRX expression (*P* < 0.0001) (Figure 3F). In addition, low ATRX expression was positively correlated with tumor type (recurrent) (*P* < 0.0046), 1p/19q non-codeletion (*P* < 0.0001), IDH status (mutant) (*P* < 0.0001), and subgroups of glioma with LGG and GBM (Figures 3G–J). Next, IHC results (Figure 3K) obtained from the HPA database showed that ATRX had low or moderate staining in glioma tissues but high intensity in normal brain tissues and that it was mainly localized in the nucleus of cells.

Next, we transfected SHG44 cells with miR-1269a inhibitor and U251 cells with miR-1269a mimic to evaluate the mRNA (Figure 3L) and protein expression (Figure 3M) levels of ATRX. As displayed in these figures, the expression of ATRX was markedly increased with miR-1269a inhibitor in SHG44 cells (*P* = 0.0045) and greatly decreased with miR-1269a mimic in U251 cells (*P* = 0.0009). Dual-luciferase reporter assay containing either the wild-type or mutated 3′-UTR of ATRX mRNA was performed to confirm whether ATRX is a direct target of miR-1269a. The results showed that, in the ATRX wild-type group, the luciferase level of the miR-1269a mimic group was significantly lower than that in the control group (*P* = 0.002), and the level in the miR-1269a inhibitor group was remarkably higher than that in the control group (*P* = 0.0297). In contrast, in the ATRX-mutated group, the levels of luciferase activities did not significantly change. These results indicated that ATRX is a direct target of miR-1269a (Figure 3N).

### ATRX Overexpression Reversed the Suppressive Effects of miR-1269a on Proliferation and Apoptosis in Glioma Cell Lines

**Figure 4:**
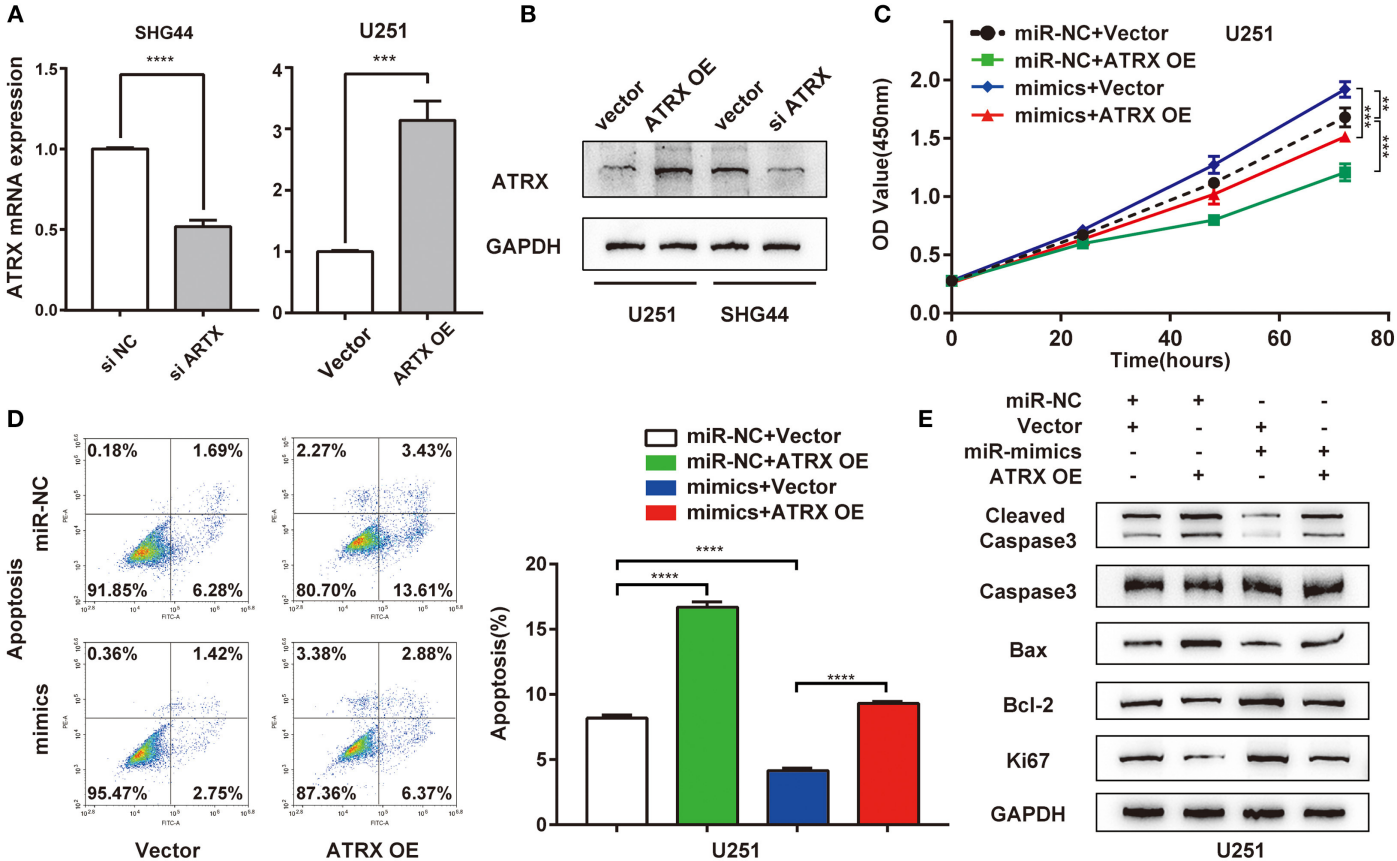
ATRX overexpression reverses the suppressive effects of miR-1269a on proliferation and apoptosis in glioma cell lines. **(A,B)** Transfection efficiency of ATRX overexpression or knockdown in U251 or SHG44 was determined by qPCR and Western blot. **(C)** Cell proliferation was determined via the CCK8 assay. **(D)** Cell apoptosis was determined via the flow cytometry assay. **(E)** The protein expression levels of proliferation (Ki67) and apoptosis (cleaved caspase 3, caspase 3, Bax, and Bcl-2) were detected using Western blot. ***P < 0.001, and ****P < 0.0001.

To further determine whether miR-1269a exerted its function via ATRX, rescue experiments were performed by transfection of a plasmid overexpressing ATRX into U251 cells treated with miR-1269a mimic. First, we transfected a plasmid overexpressing ATRX into U251 cells and si-ATRX into SHG44 cells. RT-qPCR and Western blot were used to evaluate the transfection efficiency (Figures 4A,B). CCK8 assay was performed to evaluate the cell proliferation. The results showed that, compared with the miR-NC+ vector group, overexpression of ATRX could downregulate cell viability (*P* < 0.0001). In addition, compared with the miR-1269a– mimic+ vector group, the upregulated cell viability could be reversed by transfection with ATRX-overexpressing plasmid (*P* < 0.0001; Figure 4C). A similar finding could also be discovered for the apoptosis phenotype (Figure 4D). ATRX overexpression alone could promote apoptosis, and miR-1269a mimic alone could inhibit apoptosis, whereas upon cotransfection with plasmids encoding miR-1269a mimic and plasmids overexpressing ATRX, the apoptosis rate was increased, indicating that ATRX could reverse the inhibitory effect of miR-1269a. Furthermore, Western blot assay showed that the levels of Ki67, cleaved caspase3, caspase3, Bax, and Bcl-2 were recovered in U251 cells after cotransfection with miR-1269a mimic and ATRX overexpression (Figure 4E) compared with that in the miR-1269a– mimic+ vector group. Taken together, these results suggested that miR-1269a promotes proliferation and inhibits apoptosis by targeting ATRX.

### miR-1269a Promotes Glioma Tumorigenesis *in vivo*

**Figure 5:**
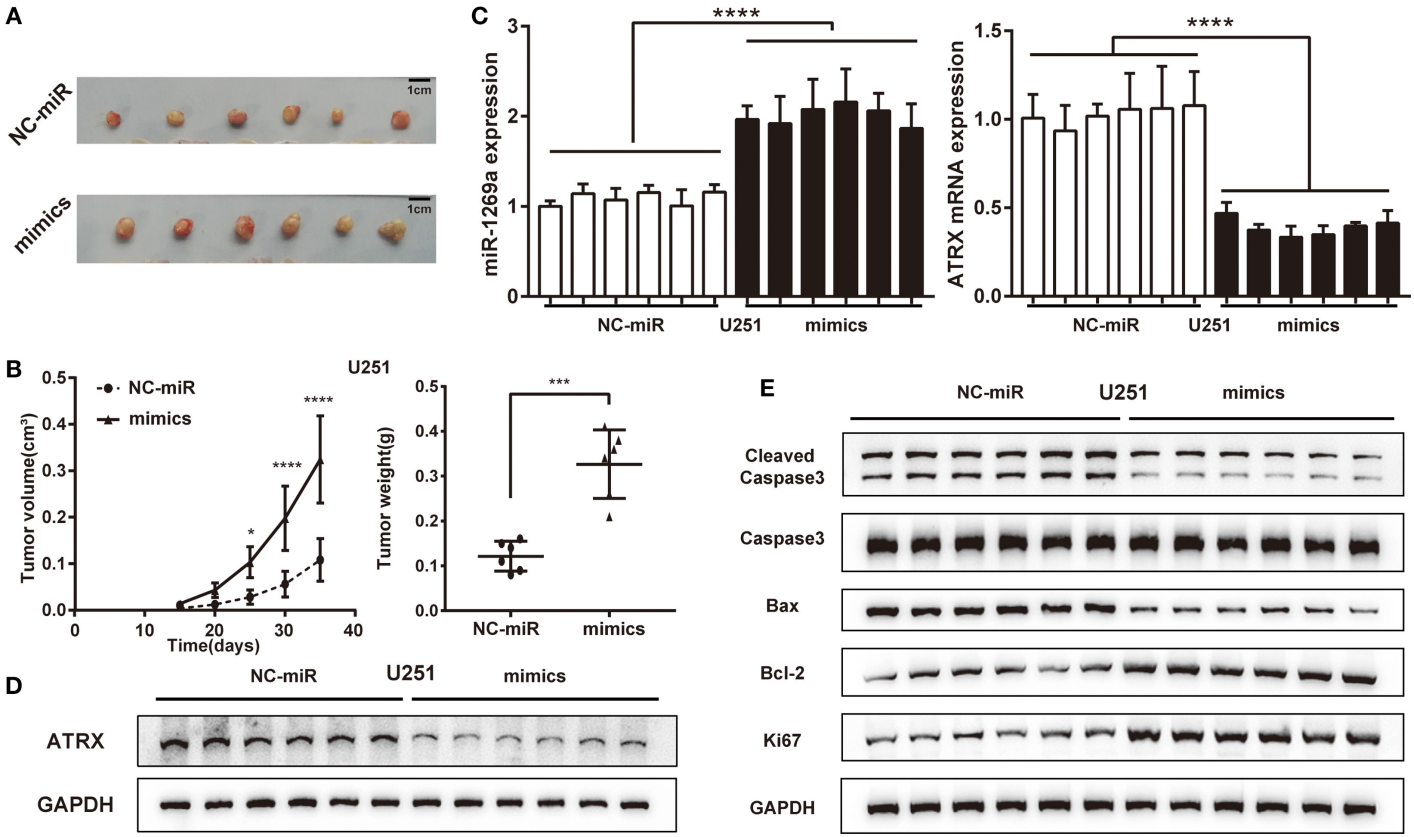
miR-1269a promotes glioma tumorigenesis *in vivo*. **(A)** A subcutaneous xenograft tumor model was used to observe the effect of U251+pLVX-miR-1269a-mimics on tumor growth. **(B)** Tumor volume and tumor weight were measured after the injection of pLVX-miR-1269a-mimics. **(C)** The relative expression levels of miR-1269a and ATRX in the xenograft tumor were detected qPCR and Western blot. **(D,E)** The protein expression levels of proliferation (Ki67) and apoptosis (cleaved caspase 3, caspase 3, Bax, and Bcl-2) in xenograft tumor were detected via Western blot. *P < 0.05, ***P < 0.001, and ****P < 0.0001.

To investigate the tumorigenesis activity of miR-1269a in glioma, we evaluated the effect *in vivo* by using a U251 xenograft tumor model (Figure 5A). The results showed that miR-1269a mimic significantly promoted tumor growth. Tumors with miR-1269a– mimic were larger in size (Figure 5B, day 25, *P* = 0.0184; day 30, *P* < 0.0001; day 35, *P* < 0.0001) and heavier in weight (*P* = 0.0001; Figure 5B) than control tumors. Moreover, qPCR assay was used to confirm the efficiency of miR-1269a mimics (Figure 5C) and the inhibitory effect of ATRX (Figure 5E). Western blot assay was used to detect the expression of ATRX protein in tumor tissues (Figure 5D) and the biomarkers of proliferation and apoptosis (Figure 5E). The results showed that the expression level of ATRX was deregulated, the proliferation level was upregulated, and apoptosis was downregulated in the miR-1269a mimic group compared with those in the control group. These results suggest that miR-1269a promotes glioma cell growth *in vivo*.

## Discussion

GBM is one of the most malignant tumors with poor prognosis and A high recurrence rate worldwide, despite the establishment of additional treatments including radiotherapy and chemotherapy (2, 14). miRNAs have been identified to be of great importance in the proliferation, invasion, migration, and development of glioma cells (15, 16).

In this study, we first analyzed differentially expressed miRNAs in two independent GSE databases and found miRNAs related to survival. Then, we obtained miR-1269a for our further experiment. This finding was also validated in another database, GSE32466, a result that revealed that miR-1269a expression was upregulated in paired recurrent GBM tissues. Previous studies showed that miR-1269a acts as a tumor promoter by targeting transforming growth factor β in colorectal cancer (4), tp53 and caspase-9 in lung cancer (6), FOXO1 in hepatocellular carcinoma (5), and RASSF9 in gastric cancer (7). Taken together, these observations suggest that miR-1269a might play the role of a tumor promoter through promoting the proliferation and metastasis of cancer cells. However, the expression and functional roles of miR-1269a in glioma are not clear. In our study, we determined the effect of miR-1269a on cell proliferation and apoptosis by measuring the expression of biomarkers and related assays. Our data illustrated that the expression of miR-1269a was higher in both cases described in databases and glioma cell lines, and it was highly associated with poorer prognosis. Moreover, the inhibition of miR-1269a significantly decreased the proliferation and increased the apoptosis *in vitro* and *in vivo*, whereas its overexpression had the opposite effects.

ATRX, one of the critical molecular biomarkers guiding the classification and diagnosis of glioma (17), plays a vital role in chromatin remodeling and the maintenance of genome and telomere stability (18, 19). ATRX loss-of-function mutations occur in about 75% of World Health Organization grade II and III astrocytoma and IDH-mutated secondary GBM cases. Besides, most LGGs with P53 and IDH1 mutations are also accompanied by ATRX loss, indicating that ATRX may be involved in the development of LGG (20). The loss of ATRX promotes tumor growth and damages non-homologous end-linked DNA repair of gliomas (21). ATRX inactivation outlines the characteristic phenotypes of glioma-derived cells (22). Moreover, it was reported that ATRX complex may contribute to temozolomide resistance in glioma (23). In our study, we illustrated that low ATRX is associated with poor characteristics (recurrence, 1p/19q non-codeletion) and prognosis of glioma patients in CGGA datasets; however, ATRX may function differently combined with other pathological characteristics such as TP53, IDH1, and TERT (17, 18); further research is still needed. Moreover, ATRX is a target gene responsible for the oncogenic role of miR-1269a in glioma. miR-1269a mimic significantly reduced the ATRX level *in vitro* and *in vivo*, whereas its overexpression had the opposite effects. In addition, the overexpression of ATRX could reverse the miR-1269a–induced cell proliferation, increasing apoptosis *in vitro*. Furthermore, we showed that the relative luciferase activity is significantly decreased in the reporter gene in 293t cells cotransfected with the miR-1269a mimic and ATRX-wt, but not in ATRX-mut. These results suggested that ATRX is a target of miR-1269a and then negatively regulates its expression. Finally, we found that ATRX overexpression could reverse the suppressive effects of miR-1269a overexpression on the proliferation and invasion of glioma cells. Therefore, our results indicate that ATRX is a direct and functional downstream target of miR-1269a.

However, a limitation should be noted. The orthotopic glioma xenograft model would better recapitulate human glioma biologically than subcutaneous xenograft model. Further investigation with orthotopic xenotransplantation is still required to validate the present results *in vitro*.

Collectively, our findings demonstrated that miR-1269a expression is significantly increased in glioma cells. miR-1269a promotes the proliferation and arrest apoptosis of glioma cells. Moreover, we verified for the first time that ATRX is a downstream target of miR-1269a and mediates its biological functions in glioma. These results suggested that miR-1269a may serve as a novel prognostic marker and that the miR1269a/ATRX axis might provide new insights into the molecular mechanisms underlying the progression of glioma.

## Data Availability Statement

The datasets analyzed for this study can be found in The Cancer Genome Atlas database (https://portal.gdc.cancer.gov/), Chinese Glioma Genome Atlas (CGGA, http://www.cgga.org.cn/), Gene Expression Omnibus database (GEO, https://www.ncbi.nlm.nih.gov/geo/) and the Human Protein Atlas (https://www.proteinatlas.org).

## Ethics Statement

The animal study was reviewed and approved by Institutional Animal Welfare and Ethics Committee, China-Japan Friendship Hospital, Beijing, China.

## Author Contributions

YZ, QW, LZ, and YY study concept and design, critical revision of the manuscript for important intellectual content. YZ, QW, and NL conducted experiments, acquisition of data, and statistical analysis. JL, HR, and XS material and technical support. All authors read and approved the final manuscript.

## Conflict of Interest

The authors declare that the research was conducted in the absence of any commercial or financial relationships that could be construed as a potential conflict of interest.
